# The Development of an Enhanced Palliative Care Pharmacy Service during the Initial COVID-19 Surge

**DOI:** 10.3390/pharmacy9040196

**Published:** 2021-12-09

**Authors:** Jaquie Hanley, Maureen Spargo, Joanne Brown, Julie Magee

**Affiliations:** 1 Antrim Area Hospital, Northern Health and Social Care Trust, BT41 2RL Antrim, Ireland; joanne.brown@northerntrust.hscni.net (J.B.); julie.magee@northerntrust.hscni.net (J.M.); 2 Medicines Optimisation and Innovation Centre, Antrim Area Hospital, Northern Health and Social Care Trust, BT41 2RL Antrim, Ireland; maureen.spargo@northerntrust.hscni.net

**Keywords:** pharmacy, palliative care, end-of-life care, COVID-19, service development

## Abstract

The Northern Health and Social Care Trust developed an enhanced palliative care pharmacy service for acute inpatients within a large hospital in Northern Ireland during the initial COVID-19 surge. By training additional staff, there was an opportunity to increase service provision, utilising palliative care pharmacy skills to undertake activities such as the symptom management of patients, appropriate management of medicines, improved access to medicines, advice for other healthcare professionals, and supporting discharge from the hospital. The data collected showed a mean of 6.8 interventions per patient, and that, irrespective of the demand resulting from the COVID-19 pandemic, the palliative care pharmacy team had a role in improving the quality of care for palliative and end-of-life patients. Subsequent data analysis also demonstrated associated cost saving and the potential for the palliative care pharmacy team to reduce the length of stay at the hospital.

## 1. Introduction

In 2004, palliative care was defined, by the National Institute for Health and Care Excellence (NICE), as “the active holistic care of patients with advanced, progressive illness”, with the aim of achieving the best quality of life for patients and those important to them. This includes management of pain and other symptoms, and the provision of psychological, social and spiritual support [1]. The World Health Organization (WHO) also recognises the need for palliative care as part of people-centred health services, stating that it is a “global ethical responsibility” to relieve health-related suffering, irrespective of the cause [2].

Medicine use plays a significant role in the management of symptoms for palliative and end-of-life care (PEOLC) patients, and polypharmacy is common among this group of patients. The management of current and previous diagnoses, side effects of treatments, use of high-risk and highly specialist medicines, prescriptions aimed at anticipating future symptom management needs, and practicalities of individual needs can all lead to complex medicine management issues.

Pharmacists are well placed to support the medicine needs of PEOLC patients. The NICE guidance on cancer services (2004), regarding improving supportive and palliative care for adults with cancer, recommends that specialist palliative care teams should include expertise provided by pharmacists, and the NICE quality standard for end-of-life care for adults (QS13) (2017) specifically notes that pharmacists should be part of a multidisciplinary workforce able to provide high-quality care and support for people approaching the end of their life [3,4]. In addition, while not specific to PEOLC, the Northern Ireland Medicines Optimisation Quality Framework (2015) recommends that patients on specialist medicines should have “access to a healthcare professional for appropriate advice and tailored, reliable information and support” [5].

It is well established that specialist palliative and end-of-life care delivered through a dedicated multi-professional team can improve the quality of care that patients receive [6,7,8,9]. Published literature recognizes the valuable role pharmacists play in delivering high-quality palliative care. Bartlett and Seager (2012) and Edwards et al. (2021) describe the role of pharmacists in hospices in England, the United Kingdom (UK) [10,11]. Several articles from the United States (US), Canada, Australia, and Europe consider the activities of the pharmacist in delivering palliative care services in a variety of inpatient and outpatient settings [12,13,14,15,16,17,18,19,20]. However, there is limited evidence quantifying the impact of the clinical role of the pharmacist. In 2019, Lehn et al. compared the impact of a palliative care pharmacist who was part of a US hospital multidisciplinary team with a similar team in another hospital with no pharmacist [21]. There were statistically significant differences in the preventable adverse drug events between the two settings, and between the activities of the palliative care pharmacist and the non-palliative care pharmacist in a single hospital.

The COVID-19 pandemic presented a number of challenges to palliative care services across the UK. There were concerns about having sufficient resources (e.g., staffing, medicines, equipment, etc.) to cope with the potential deaths, and whether those resources would be available in the most appropriate setting.

### Outlining the Need for Service Development

The Northern Health and Social Care Trust (NHSCT) has a population of around 480,000, and is one of five health trusts in Northern Ireland. A dedicated, multi-professional team provides input to patients in a bespoke 12-bedded palliative care inpatient unit, and a nurse-led hospital specialist palliative care team supports palliative care patients in the acute setting. The hospital-based palliative care nursing team can seek advice from senior specialist palliative care medical staff, but there is no dedicated support from the extended specialist palliative care multi-professional team for the general wards.

Limited information was available on the needs of the PEOLC inpatients in the NHSCT beyond those reviewed by the palliative care nursing team or admitted to the specialist inpatient unit. It is reasonable to assume that many of the acute inpatients with palliative conditions will have symptoms or other issues that could benefit from the input of a specialist palliative care team.

At the NHSCT, the specialist palliative care pharmacist is frequently contacted to advise staff elsewhere in the trust, regarding the management of inpatients with palliative and end-of-life care needs—these queries increased during the first surge of the COVID-19 pandemic. This strongly indicated that there was a need for a palliative care pharmacy service beyond the dedicated inpatient unit. In the absence of such a service, it is highly likely that opportunities to improve the quality of palliative and end-of-life care were being missed, with regards to the management of medicines, and the quality and safety of medicine prescribing.

This paper presents the evidence gathered during the evaluation of a rapidly developed palliative care pharmacy service for acute inpatients during the initial surge of the COVID-19 pandemic.

## 2. Materials and Methods

### 2.1. Details of the Service

As part of the response to the anticipated COVID-19 pandemic, the specialist palliative care pharmacist (J.H) was allocated to a large acute hospital on a full-time basis. Experienced pharmacists (J.B and J.M) were redeployed to work alongside the specialist pharmacist to develop a dedicated palliative care clinical pharmacy service to the acute wards.

The redeployed pharmacists underwent intensive training in palliative care pharmacy, focusing on end-of-life care, through an accelerated ‘Teach and Treat’ model (i.e., enhancing clinical skills of pharmacists through demonstration and clinical supervision by an experienced pharmacist, to develop competence and confidence in managing a group of patients) supported by case-based discussion and by a range of educational resources [22].

Patients were identified through direct referrals from ward pharmacy, medical or nursing staff or daily liaison and referrals from the hospital palliative care nurses. The pharmacists were all independent prescribers and therefore were able to directly make changes to the prescription charts. Initially, the service focused on the end-of-life care needs of COVID-19 patients; however, the service was increasingly involved in the review of non-COVID PEOLC patients according to the demand. Due to this increased demand and to facilitate timely symptom management and patient review, the palliative care clinical pharmacy team extended their service from five to six days per week. After the initial surge, the demand for PEOLC input for COVID-19 patients across the trust decreased and the redeployed pharmacists gradually returned to their substantive posts. By June 2020, there was just one pharmacist delivering the palliative care clinical pharmacy service.

### 2.2. Aims and Objectives

The primary aim of the research was to evaluate the impact of the new inpatient palliative care clinical pharmacy service on the quality of care delivered to PEOLC patients in the acute hospital setting. Key objectives identified were as follows: (a) describe the activities conducted by the pharmacist, (b) describe and quantify the impact of a palliative care pharmacist on the quality of care of PEOLC patients, and (c) evaluate the potential associated economic advantages of the service for the organization.

### 2.3. Study Design

The palliative care pharmacy service maintained a case load of patients during the initial surge of the pandemic. Data were collected on every patient interaction over three months. Basic demographic information, relevant medical history, laboratory results and current issues, and the details of any interventions made by the pharmacists were documented. Details were recorded on those patients who were being treated as ‘end of life’ and/or for COVID-19.

#### 2.3.1. Palliative Care Pharmacist Activities and Quality of Care

Information about the types of interventions made by the pharmacists during the data collection period were summarised and grouped into themes to describe the types of activities undertaken.

To determine the impact on quality of care, all interventions carried out by the pharmacists were reviewed. The quality of the interventions was then assessed using the Eadon intervention ranking scale (see Table 1) [23]. This scale grades the quality of pharmacist interventions from 1 to 6. Interventions were first graded by a pharmacist who was independent of the palliative care service, and then verified by a second pharmacist more familiar with the service. The mean number of interventions per patient and the number and proportion of each grade of intervention were determined.

#### 2.3.2. Economical Assessment

To assess the economic impact of the service, potential cost avoidance following pharmacist intervention and length of stay at the hospital were measured.

##### Cost Avoidance

The Sheffield University economic model (ScHARR; School of Health and Related Research) for the potential healthcare costs avoided by clinical interventions made by a consultant pharmacist was used to measure cost avoidance [24]. This model refers to all medicine-related costs. The model was established to evaluate other pharmacist-led services in Northern Ireland and is linked to the Eadon intervention ranking scale [25,26]. Each Eadon-graded intervention can be assigned an associated cost avoidance as illustrated in Table 2.

Findings from the number and grade of pharmacist inventions were used to calculate the estimated cost avoidance that resulted from the service. Firstly, the mean number of interventions made by a pharmacist per month per grade was determined. These numbers were then multiplied by the midpoint value of the ScHARR range (i.e., grade 3: GBP 3; grade 4: GBP 108; grade 5: GBP 1099) to estimate the costs saved per month per grade of intervention; for example, the mean number of grade 4 interventions per month was multiplied by GBP 108 to provide an estimate of the costs avoided per month as a result of all grade 4 interventions made by the pharmacists. The total cost avoidance of all interventions was then collated to estimate the total cost avoidance per month.

The outcome of the ScHARR cost avoidance calculations was subsequently adjusted because it is not yet established if the model is applicable to a PEOLC population. The outcomes were adjusted by only considering the cost avoidance for the proportion of patients *not* considered to be at the end of life.

The cost avoidance calculations were also used to predict potential cost savings for future services. The proportions of each grade of intervention made by the palliative care pharmacy service during the three-month data collection period were used to determine the anticipated total number of interventions one pharmacist could make per month, which was then multiplied by the midpoint value of the ScHARR range to determine potential cost avoidance for a future service comprising one pharmacist working full-time.

##### Length of Stay

To measure the impact on length of hospital stay, a sample of the population of patients who received the palliative care pharmacy service was followed up retrospectively and compared with a control sample of palliative care patients. To select a sample, 25% of patients who received the pharmacist service in June and July were selected randomly. A comparative sample was obtained from the cohort of palliative care patients who were managed by the palliative care nursing team, but did not receive the pharmacist service during the same two-month period. A sample population was used due to time constraints. The length of stay in hospital in days was extracted from computerised patient records.

## 3. Results

Data on 194 patients who received the palliative care pharmacy service between April and June 2020 were collected. The proportion of COVID-19 patients declined from 33% to 4% between April and June, and approximately two thirds (67%) of the patients were considered to be at the end of life. Table 3 describes the characteristics of the case load of patients who were managed by the team.

### 3.1. Palliative Care Pharmacist Activities and Quality of Care

A total of 1309 interventions were made by the specialist palliative care pharmacy service over three months. There was a mean of 436 interventions per month and a mean of 6.8 interventions for every patient over an average of 3.1 visits. The activities of the palliative care pharmacy team were diverse, and common themes are noted in Table 4.

The quality of 80% of the interventions was considered to be grade 4—‘significant and resulted in an improvement in the standard of care’. Grade 5 interventions accounted for 5% of all the interventions, equating to 75 separate interventions in three months that were ‘significant and prevented major organ failure or adverse reactions of similar importance’. Table 5 presents the number and grade of the interventions.

### 3.2. Economic Impact

#### 3.2.1. Cost Avoidance

The potential cost avoidance per month resulting from all the interventions made by the palliative care pharmacy team was GBP 61,824 (see Table 6). When adjusted to include the cost avoidance from only those patients who were not at the end of life (33%; Table 3), the estimated cost avoidance was GBP 20,402/month.

The potential future cost avoidance resulting from one full-time palliative care pharmacist was then estimated (see Table 7). The findings indicated that one pharmacist could review 45 patients per month. The data collected found a mean of 6.8 interventions per month. To prevent over estimation, this was rounded down to six interventions per month. The potential cost avoidance per month for one full-time equivalent pharmacist was estimated to be GBP 38,287, and following adjustment for those patients not at the end of life (33%), the cost avoidance was GBP 12,635/month.

#### 3.2.2. Length of Stay

There were 78 patients reviewed by the pharmacy service between June and July. Twenty-five percent of these patients (*n* = 20) had their length of stay in the hospital documented. One hundred and nineteen palliative patients were not reviewed by the pharmacy service, but were in hospital during the same time period. Twenty-five percent of these patients (*n* = 30) had their length of stay in the hospital documented. The data illustrated in Table 8 indicate that those palliative care patients who were reviewed by a palliative care pharmacist had a shorter length of stay compared with those patients with no palliative care pharmacist involvement (8.75 days versus 10 days). The difference was greater for those patients who were discharged from hospital (7.9 days versus 11.8 days).

## 4. Discussion

Within the NHSCT, clinical pharmacists and technicians have been delivering integrated medicine management services to all the general wards in acute hospitals for over 20 years. It is a well-established service, with the pharmacy team viewed as a vital component of high-quality ward-based care in the trust. However, prior to the pandemic, the expertise of the specialist palliative care pharmacist was only available part-time for the specialist inpatient unit, or remotely for advice. The development of this enhanced service brought the knowledge and skills of the palliative care pharmacist to patients’ bedsides in general wards. The number and quality of interventions undertaken by the palliative care pharmacy team indicate that the service had a direct benefit on the patients’ quality of care. The enhanced palliative care pharmacy service was provided in addition to established clinical pharmacy and hospital palliative care nursing services; therefore, the interventions undertaken by the palliative care pharmacy team can be viewed as additional to these ‘normal’ services, although it is noted that there was probably a small amount of overlap. It was also recognised that the data collected during this time would not be truly reflective of the service demands outside of the pandemic, but they still provide an indication that the palliative care pharmacist can have a positive impact on the quality of care for PEOLC patients in the acute inpatient setting. The activities undertaken by this team are in keeping with other published reports on the activities of palliative care pharmacists across the world [10,14,15,16,21].

The total number of patients reviewed by the team, and, subsequently, the number of interventions undertaken, declined month on month. However, rather than being due to a lack of demand, this can be attributed to a change in the availability of staff, as the ‘new’ pharmacists returned to their substantive posts. The number and trends in the grade of interventions undertaken through this new service suggest that a palliative care pharmacist has a significant role to play in the care of PEOLC patients. The percentage of end-of-life care patients remained at approximately two thirds of all the patients reviewed. However, even as the proportion of COVID-19 patients declined, the mean number of interventions per patient and the proportion of Eadon grade 5 interventions did not change. Therefore, even in the absence of COVID-19 demands, the palliative care pharmacist continued to have a positive impact on the care that PEOLC patients received in the acute setting.

The limitations of this model include the use of the Eadon grading system, as the definitions used are quite broad and are not easily applicable to the PEOLC population. While literature describing the role of the palliative care pharmacist is plentiful, there is a lack of comparative evidence on the quantity and impact of individual interventions made by palliative care pharmacists, especially in the acute hospital setting. Wilby et al. (2014) classifies the type and number of interventions undertaken by a pharmacist in a hospital in Qatar, and notes an average of 3.0 interventions per patient, but does not go so far as to rate the direct patient impact [27]. The pharmacists described in the Wilby paper are not independent prescribers, which may account for the difference in the number of interventions per patient compared to our results. It would certainly be useful to undertake similar research in other centres and geographical areas to compare with our outcomes.

The ScHARR model used to consider the economic implications of the palliative care pharmacist role does have some limitations. The model was developed in 2009 and is, therefore, likely to underestimate the current healthcare costs, and it is unclear if this model is applicable to the PEOLC population. However, its use is established within pharmacy services in Northern Ireland and was warranted in the absence of a suitable alternative, with mitigation measures employed to compensate [24,25,26]. Despite these limitations, economic analysis of the interventions demonstrates that not only does the palliative care pharmacist influence the appropriateness and safety of medicine use in this patient population, there is also likely to be an associated cost saving. This is in keeping with the findings of other recent literature, which suggest that the involvement of the palliative care pharmacist in a dedicated multidisciplinary team can lead to significant cost efficiencies, with respect to the return on investment [21].

The retrospective review of length of stay at the hospital indicates that access to a dedicated palliative care pharmacist may also have a benefit on reducing hospital stay. Recent studies based in the US have also reported that early review and intervention by a palliative care pharmacist can reduce the length of stay at the hospital [19,20]. Further work would be useful in this area, to validate these findings in the UK and Ireland, and examine the impact on the rates of emergency department attendance, readmission, and out of hours calls to primary care services after discharge. Lehn et al. (2019) also recognizes that further work would be useful in establishing the impact of the palliative care pharmacist on the length of stay at the hospital, emergency department attendance, and 30-day readmission rates [21].

## 5. Conclusions

The primary aim of this paper was to evaluate the impact of a palliative care pharmacist on quality of care for PEOLC inpatients in the acute hospital setting during the initial COVID-19 surge. The findings indicate that the specialist palliative care pharmacy team had a positive impact on the care the patients received, with respect to their medicine management issues. The results suggest that additional benefits may include substantial financial savings and reduced resource use. This evaluation has also revealed that methodological tools that can accurately measure the impact that pharmacists can have on the quality of care of palliative care patients are urgently required, in order for the evidence gaps in this field of practice to be filled.

## Figures and Tables

**Table 1:** Eadon intervention grading system [23].

Ranking	Description
Grade 1	Detrimental to patient’s well being e.g., removing drug or decreasing dose inappropriately
Grade 2	No significance to patient care e.g., endorsing generic drug names
Grade 3	Significant, but does not lead to an improvement in patient care e.g., requests for change in treatment on cost basis
Grade 4	Significant and results in an improvement in the standard of care e.g., getting omitted drugs added to kardex
Grade 5	Significant and prevents major organ failure or adverse reaction of similar importance e.g., calculation of aminoglycoside dose with impaired renal function
Grade 6	Potentially life-saving e.g., life threatening drug interaction

**Table 2:** ScHARR model of medicine-related cost avoidance [24].

Cost Avoidance ScHARR Model GBP		Eadon Criteria Ranking
Potentially Lethal	1085–2120	6
Potentially Serious	713–1484	5
Potentially Significant	65–150	4
Minor	0–6	1–3

**Table 3:** Patients seen by the palliative care pharmacy team.

Month	Total Number of Patients	Number of Patient Visits	Mean Number Visits/Patient	% COVID Patient	% End of Life Care Patient
April	81	278	3.4	33%	69%
May	68	215	3.2	16%	66%
June	45	124	2.8	4%	67%

**Table 4:** Pharmacist activities.

Common Activity Themes for Palliative Care Pharmacy Team
Reviewing and prescribing medication for the management of symptoms. Prescribing anticipatory medicines and/or syringe pumps as appropriate.
Reviewing medication and stopping medicines no longer appropriate (including stopping anticipatory medications when a patient was no longer in end-of-life care).
Adjusting medicines prescribed (i.e., change in drug, dose, frequency, route, etc.) according to individual needs (e.g., if renal or hepatic impairment was present).
Advocating for patients and prompting staff to manage symptoms appropriately (e.g., encouraging timely medicine administration).
Preparation of prescriptions, ensuring appropriate transfer of information to GP and community pharmacy.
Providing advice as needed to hospital palliative care nursing team, community teams, ward medical team, and nursing and pharmacy staff.

**Table 5:** Summary of Eadon-graded interventions.

Month	Total Number Interventions	Mean No. Interventions/Patient	Grade 3		Grade 4		Grade 5/6	
			Total	%	Total	%	Total	%
April	517	6.4	117	23	367	71	33	6
May	481	7.1	56	12	400	83	25	5
June	311	6.9	24	8	270	87	17	5
Mean *	**436**	**6.8**		**15**		**80**		**5**

* rounding to whole numbers may result in very slight variation in calculation.

**Table 6:** Estimated ScHARR cost avoidance each month for interventions by palliative care pharmacy service.

Eadon Grade	Mean Number of Interventions per Month per Grade	ScHARR Cost Avoidance (Taken as Mid-Point of Range, GBP)	Total Estimated Cost Avoidance per Month per Grade (GBP)
3	65.4	3	196
4	348.8	108	37,670
5	21.8	1099	23,958
Total Estimated Cost Avoidance			GBP 61,824/month
Total Estimated Cost Avoidance Adjusted for Patients *not* at end of life (i.e., 33%)			GBP 20,402/month

**Table 7:** Prediction of potential ScHARR cost avoidance associated with palliative care pharmacist interventions for one full-time equivalent pharmacist.

Anticipated Mean no. Patients per Month = 45 (for 1 Full-Time Equivalent Pharmacist)				
Estimated No. Interventions/Patient = 6.8 (Rounded Down to Nearest Whole Number = 6)				
Eadon Grade	% of Total Interventions/Month	Anticipated Number of Interventions per Month	ScHARR Cost Avoidance (Taken as Mid-Point of Range, GBP)	Total Potential Cost Avoidance (GBP)
3	15	40.5	3	122
4	80	216	108	23,328
5	5	13.5	1099	14,837
Total Potential Cost Avoidance				GBP 38,287/month
Total Potential Cost Avoidance Adjusted for Patients *not* at end of life (i.e.,33%)				GBP 12,635/month

**Table 8:** Impact on length of stay (LOS) for inpatients reviewed by palliative care (PC) pharmacist.

	Number of Patients Reviewed	% Patients Who Died in Hospital	Average LOS (Days)	Average LOS in Patients Discharged
Review or Intervention by PC Pharmacist	20	35%	8.75 (range 2 to 26)	7.9 (range 2 to 15)
No Review or Intervention by PC Pharmacist	27 *	48%	10 (range 2 to 29)	11.8 (range 2 to 29)

* 3 patients excluded as information was incomplete or they were identified as having pharmacist intervention.

## Data Availability

The datasets generated during and/or analyzed during the current study are available from the corresponding author on reasonable request.
